# Effects of cerebellar transcranial magnetic stimulation in different protocols on working memory: An EEG study

**DOI:** 10.1002/alz70860_097095

**Published:** 2025-12-23

**Authors:** Bo Song, Jingping Shi, Minjie Tian

**Affiliations:** ^1^ The Affiliated Brain Hospital of Nanjing Medical University, Nanjing, Jiangsu, China; ^2^ Brain Hospital of Nanjing Medical University, Nanjing, Jiangsu, China

## Abstract

**Background:**

Research increasingly shows that the cerebellum plays a key role in regulating non‐motor functions like cognition, language, and emotion, through complex interactions with brain circuits. Its synaptic plasticity and information integration abilities make it a promising target for cognitive modulation therapies. Our previous studies have shown that 5Hz rTMS can improve cognition, but the iTBS protocol, more commonly applied to the brain, induces stronger oscillatory activity and better cognitive improvements. This study aims to explore the effects of different TMS protocols on working memory when applied to the cerebellar Crus II region, and investigate the underlying neural mechanisms.

**Method:**

Sixty healthy adults participated in this study, with the right cerebellar Crus II region as the target for intervention, localized using MRI and neuronavigation. Participants were randomly assigned to one of three groups: 5Hz rTMS, iTBS, or sham stimulation. Before and after the intervention, all participants performed a 2‐back working memory task, and EEG signals were continuously recorded using a 71‐channel system. Behavioral measures, event‐related potential (ERP) amplitudes, time‐frequency changes, and brain network topology were compared within and between groups.

**Result:**

Both the 5Hz rTMS and iTBS groups showed significantly shorter reaction times than the sham group, with the 5Hz rTMS group showing a more pronounced improvement. Neurophysiological data revealed increased P150 amplitudes in both active groups, with the 5Hz rTMS group showing significantly higher P150 amplitudes than the iTBS and sham groups. Time‐frequency analysis showed that 5Hz rTMS enhanced θ and α oscillations, while iTBS and sham groups did not. The 5Hz rTMS group also had significantly higher α oscillatory activity compared to iTBS and sham groups. Resting‐state brain network analysis showed increased global efficiency in the θ frequency band in both active groups, with no differences between them.

**Conclusion:**

The 5Hz rTMS intervention to the cerebellar Crus II region more significantly enhanced brain oscillatory activity and improved working memory performance compared to the iTBS intervention. These findings provide insights into optimizing parameters for cerebellar TMS in the improvement of memory functions.

